# Discrepancy between Self-Reported and Urine Cotinine-Verified Environmental Tobacco Smoke Exposure among Rural Pregnant Women in China

**DOI:** 10.3390/ijerph15071499

**Published:** 2018-07-16

**Authors:** Xia Xiao, Yan Li, Xiaoxiao Song, Qinghua Xu, Siwei Yang, Jie Wu, Edmund Seto

**Affiliations:** 1 School of Public Health, Kunming Medical University, 1168 Chunrongxilu Road, Yuhua Neighborhood, Chenggong, Kunming, Yunnan 650500, China; xxkmyn@gmail.com (X.X.); songxiaoxiao@kmmu.edu.cn (X.S.); xqhyn2018@126.com (Q.X.); ky_axwrysw@126.com (S.Y.); wujiesher@126.com (J.W.); 2 School of Public Health, University of Washington, Box 357230, Seattle, WA 98195, USA; eseto@uw.edu

**Keywords:** environmental tobacco smoke (ETS), self-report, cotinine, pregnant women

## Abstract

Prenatal exposure to environmental tobacco smoke (ETS) is the most modifiable risk factor associated with adverse child-health outcomes. However, few longitudinal studies are implemented to compare the rates of discrepancy between self-reported (SR) and urinary cotinine (UC)-verified ETS exposure during the three trimesters of pregnancy, especially in rural areas. The objectives of this study were to assess the discrepancy between SR and UC-verified ETS exposure among rural women employing three measures throughout pregnancy, and to explore predictors related to these differences. This study used a prospective prenatal cohort consisting of 420 pregnant women whose ETS exposure was entirely evaluated by both SR and UC verification across three trimesters of pregnancy. Environmental tobacco exposure was assessed via SR verification, and was validated using the limit of detection for UC. The discrepancy rates were determined for each trimester. Multivariate logistic regression was used to assess the predictors associated with these differences. The discrepancy rates between SR and UC verification were 25.2%, 17.1%, and 20.5% (first, second, and third trimester, respectively). The highest inconsistency occurred in the first trimester. After adjusting for confounding factors, the following variables were found to have statistically significant associations with the discrepancy rate between SR and UC-verified ETS exposure: the number of smokers in the family and household income for all three trimesters, township site for the second and third trimester, and gravidity for the last trimester. The SR rate of ETS exposure among rural pregnant women is underreported, while the UC-verified rate is higher. More smokers in the family and gravidity may increase the risk of ETS exposure for pregnant women. Biochemical validation is warranted throughout pregnancy for the adoption of home-smoking bans and the promotion of community-based smoke-free programs.

## 1. Introduction

Prenatal passive smoking is a prevalent environmental exposure that is associated with adverse infant and childhood health outcomes [1,2]. Passive smoking is defined as the combination of secondhand smoke (SHS) and thirdhand smoke (THS) exposure [3]. SHS is “the combination of smoke emitted from the burning end of a cigarette or other tobacco products and the smoke exhaled by the smoker” [4], and SHS exposure results from the involuntary inhalation of sidestream and exhaled mainstream smoke [5]. In contrast, THS is derived from SHS, whereby the residue from tobacco smoke persists on the clothing and hair of smokers, on environmental surfaces, and in dust long after a cigarette is extinguished [6], resulting in the involuntary inhalation, ingestion, or dermal uptake of THS pollutants in the air, in dust, and on surfaces [5]. Passive-smoke exposure, which is currently defined as environmental tobacco smoke (ETS) exposure, is particularly concerning for pregnant women, and was shown to cause preterm delivery and spontaneous abortion [1,7], low birth weight, sudden infant death syndrome, childhood asthma, and respiratory illness [8,9]. China is the world’s largest producer and consumer of tobacco, with over 350 million smokers [10]. Based on the 2010 Chinese National Survey, the prevalence of passive smoking self-reported by nonsmokers aged 15 or above was 72.4% [11,12], with the prevalence being higher for women and in rural areas, compared with that for men and in urban areas, respectively. Moreover, one cross-sectional study, which evaluated urinary cotinine concentration in Chinese pregnant women, found that the rate of detection of urine cotinine was as high as 87%, indicating that pregnant women were at high risk of being exposed to ETS [13].

Reliable information regarding ETS exposure is necessary for providing appropriate medical advice regarding prenatal care and planning follow-up visits. The determination of passive smoking is usually self-reported (SR) information, or via measures of nicotine metabolites in human fluids such as urine, saliva, and blood [14]. Self-reported surveys are generally used in studies evaluating the rate of smoking due to their convenience and economic feasibility. Previous studies primarily investigated the accuracy of SR surveys of active smoking, most often through the biochemical validation of cotinine [15], in populations that were pressured to abstain from cigarette smoking for medical or social reasons, such as pregnant women [16,17,18], teenagers [19,20,21], and those undergoing smoking cessation therapy [22,23]. Although comparative studies assessed cotinine levels to validate self-reported ETS exposure among urban/general populations using a cross-sectional study design, few longitudinal studies exist that compare the rates of SR and urinary cotinine (UC)-verified ETS exposure among rural pregnant women throughout pregnancy.

This study sought to examine SR and UC-verified ETS exposure among rural pregnant women across each trimester, in an effort to assess the discrepancy between the rates of SR and UC-verified ETS exposure, and to explore predictors related to these differences.

## 2. Materials and Methods

### 2.1. Study Design and Population

All data were collected from pregnant women participating in the Xuanwei Study, an ongoing prospective prenatal cohort study in rural China designed to examine exposure to environmental toxins, birth outcomes, and child growth and development in the community. From July 2014 to July 2017, pregnant women who were confirmed to be in the first trimester of their pregnancy were recruited from 20 village clinics within two townships in Xuanwei County, located in the Yunnan Province. Township 1, located in the northern region of the county, is known for its coal, grain, and pig industries, while Township 2 is located in the southwest region of the county, and its primary industry is agriculture, including the production of tobacco and rice. Any individuals with missing values for SR or UC verification at any time of measurement were removed. Ultimately, the pregnant women involved in the analysis fully completed the appropriate exposure evaluation during the first, second, and third trimester. A total of 420 pregnant women from the cohort study participated in the repeated survey of both SR and UC-validated ETS exposure.

The institutional review boards (IRBs) of Kunming Medical University and Yunnan Maternal and Child Hospital approved this study. Additionally, the Maternal and Child hospital in Xuanwei County and the local township hospital’s IRB were involved in the oversight of this study. All pregnant women provided written informed consent prior to enrollment in this study. Eligibility criteria included <19 weeks gestation, non-smoker, local resident for at least two years in one of the 20 villages, intention of continuing prenatal care and of delivering at the collaborating obstetric practice, negative HIV status, and not receiving seizure, thyroid, or chemotherapy/radiation medications. We recruited pregnant women who were living in homes in one of the two townships, who were examined by the village doctors to determine whether they were eligible and interested in participating in the Xuanwei study. Our analyses were restricted to singleton infants. Participants completed the surveys on paper documents in the first trimester (from pregnancy confirmed to 12 weeks gestation), second trimester (17–23 weeks gestation), and third trimester (32–35 weeks gestation), and they were given a modest compensation after having completed data collection at the three time points, and having given birth. Moreover, participants also provided midstream urine samples of 20 cc or more in a disposable container at the same visit during their pregnancy. All urine samples were stored at −25 °C until they were transported to the laboratory of the Kunming Medical University for analysis, where they were stored at −80 °C.

### 2.2. Assessment of Environmental Tobacco Smoke (ETS) Exposure Based on Self-Reports

In each of the pregnancy trimesters, self-reported ETS exposure was collected via a questionnaire during face-to-face interviews. The participants were asked the following questions: “Are there smokers living with you?”, “Did anyone, including family members and visitors, smoke cigarettes near you (within 3 m) when you were at home during the past 7 days?”, and “Did anyone smoke cigarettes near you (within 3 m) when you were outside the home during the past 7 days?”. They had the following two options as answers: (1) “no”, and (2) “yes, number of cigarettes_____”. Those who answered “no” to both of the above questions were coded as “non-ETS exposure”; those who answered “yes” to either of the above questions were coded as “ETS exposure”.

### 2.3. Urine Biomarkers of ETS Exposure

Urine samples were analyzed for cotinine, a biomarker of nicotine exposure, using high-performance liquid chromatography/tandem mass spectrometry (HPLC-MS/MS) [24]. The transition ions for cotinine monitored in this work were 177/98.2 and 177/80.1. The limit of detection (LOD) for cotinine was 0.08 ng/mL. The samples were analyzed using multiple reaction monitoring, and concentrations were calculated from the ratios of native and labeled ions within the samples compared to a five-point calibration curve.

### 2.4. Demographic Information

The study used face-to-face questionnaires to obtain the participants’ demographic information. Demographics included age (in years), race/ethnicity, occupation and education (with three options, ranging from “six years and below” to “10 years and above”), and household income (with five options, ranging from “no response” to “50,001 CNY or more”) of the pregnant women. Other personal factors included period of pregnancy, women’s awareness of passive smoking (participants were asked “Have you heard of passive smoking?”; those who answered “no” were coded as “non-perception of ETS”, and those who answered “yes” and explained it as non-smokers inhaling smoke from the smoker were regarded as “perception of ETS”), number of pregnancies (gravidity), and numbers of smokers in the family. Additionally, the age, race, occupation, and education of the husbands of the participants were also considered.

### 2.5. Statistical Analysis

Descriptive statistics, including means, standard deviations, and frequency distributions, were used to summarize personal characteristics and ETS-exposure indicators. In order to verify the inconsistency between SR and UC verification, we performed chi-squared tests to distinguish between variables that were significantly different across groups, whereby the two values were inconsistent (*p*-value < 0.05). Multivariate logistic regression was used to assess the maternal and family factors associated with any inconsistency between SR and UC verification rates in each trimester. Data analysis was done using the STATA Stata/SE ver 14.0 software (StataCorp LP, College Station, TX, USA), with an alpha level of 0.05. 

## 3. Results

### 3.1. Pregnant Women Characteristics

Characteristics of the participants are described in Table 1. Out of a total of 420 participants, 181 (43.1%) were from Township 1, and 239 (56.9%) were from Township 2. The average age was 24.7 years (SD = 5.0). Most participants were between 20 and 25 years of age (50.2%), were of Han ethnicity (84.8%), were farmers (80.0%), and had at least a middle-school education (66.4%). Moreover, all participants were married or otherwise partnered. The majority of husbands of the participants were of Han ethnicity (87.9%), were farmers (69.4%), and had at least a middle-school education (75.4%). Nearly 63% of the respondents had an annual household income of less than CNY 30,000 (about US$ 4677); therefore, they belonged to the lowest socio-economic class in China, and, under Chinese regulation, they were designated as “needy families” (the threshold is CNY 30,000).

### 3.2. Discrepancy between SR and UC-Verified ETS Exposure

A comparison of SR and UC-verified ETS exposure is presented in Table 2. There were 323 (76.9%) SR cases of exposure to ETS in the first, 350 (83.3%) in the second, and 338 (80.5%) in the third trimester. The rate of UC-verified ETS was 97.4% in the first trimester, 99.1% in the second trimester, and 98.6% in the last trimester of pregnancy. Therefore, the discrepancy rates between SR and UC-verified ETS exposure were 25.2%, 17.1%, and 20.5% for the first, second, and third trimesters, respectively. These rates differed significantly from each other at the 0.05 level, and the highest inconsistency occurred in the first trimester. Moreover, the percentage of UC-verified cases of ETS exposure tended to be higher than that of SR cases among the pregnant women who lived in Township 2 and had lower educational levels, non-famer husbands, awareness of passive smoking, more gravidity (pregnancy number of three), and more smokers (smoker number of two or three) in the family, determined by means of analysis of the discrepancy between SR and UC verification relative to the study variables. The discrepancy rates across households with different annual incomes also showed a statistically significant difference. Univariate analysis did not reveal a statistically significant correlation between age, ethnicity, occupation of pregnant women, or husbands’ education level, and the discrepancy rate.

### 3.3. Predictors Associated with the Discrepancy between Rates of Self-Reported and Urinary Cotinine-Verified ETS Exposure

We analyzed variables for association with inconsistency between SR and UC-verified rates using multivariate logistic regression (Table 3). Though the variables, “age of the pregnant women” and “awareness of passive smoking”, were found to have no statistically significant associations in the final logistic regression model in this study, age was related to the number of pregnancies [25], and the awareness of passive smoking was related to self-reported ETS exposure [26] based on previous studies. After adjusting for both variables, household income, number of smokers in the family, township site, and gravidity were found to be associated with the discrepancy between SR and UC-verified ETS exposure as pregnancy progressed. In the early stages of pregnancy, we could expect the odds of discrepancy among participants with an annual household income of 50,001 CNY and above to be 0.11 times the odds of discrepancy among participants earning less than 15,000 CNY annually (adjusted odds ratio (aOR), 0.11; 95% confidence intervals (CIs), 0.04–0.36). Participants who had some smokers in the family had an aOR of 7.14 (95% CIs, 2.64–19.31) for one smoker, and an aOR of 24.22 (95% CIs, 8.35–70.26) for two and three smokers, compared with participants who had no smokers in the family. For the second trimester, pregnant women living in Township 1 were more likely to have an SR/UC discrepancy than those in Township 2, with an aOR of 3.72 (95% CIs, 1.56–8.88). Participants with an annual household income of 15,001–30,000 CNY and 30,001–50,000 CNY were 2.40 (aOR, 2.40; 95% CIs, 1.11–5.21) and 5.09 times (aOR, 5.09; 95% CIs, 1.77–14.68) more likely to have an SR/UC discrepancy, respectively, than those earning less than 15,000 CNY annually. Participants who had some smokers in the family had an aOR of 5.01 (95% CIs, 1.94–12.96) for one smoker, and an aOR of 12.49 (95% CIs, 4.45–35.05) for two and three smokers, compared with participants who had no smokers in the family. In the last trimester, pregnant women from Township 1 were still more likely to have an SR/UC discrepancy than those in Township 2, with an aOR of 3.51. We could also expect the odds of discrepancy among participants with an annual household income of 50,001 CNY and above to be 0.28 times that among participants earning less than 15,000 CNY annually (aOR, 0.28; 95% CIs, 0.81–0.98), similar to the first trimester. Participants with a gravidity of three and above were 2.72 times more likely to show discrepancy between SR and UC-verified rates than participants with a gravidity of one (aOR, 2.72; 95% CIs, 1.08–36.81). Participants who had two or three smokers in the family had an aOR of 6.10 (95% CIs, 2.78–13.39), compared with participants who had no smokers in the family. The occupations of their husbands did not show significant correlation for any trimester.

## 4. Discussion

In this study, we found that biologically verified rates of ETS exposure were higher than the SR rates of ETS exposure throughout pregnancy. The result indicated that, regardless of trimester, more than 15% of pregnant women with actual exposure to ETS may not perceive themselves as passive smokers in prenatal care, especially in the first trimester. These rates of discrepancy for ETS exposure were lower than those observed in similar studies conducted among pregnant women [16]. The possible reasons are that those studies included both active and passive smokers, and had cotinine measures above the minimum threshold for tobacco use among pregnant women who reported a non-smoking or quit status [16]. Kristin [16] and Wigginton [27] indicated that a higher misclassification rate among pregnant women was associated with the stigma related to smoking during pregnancy. For example, pregnant women who smoked were likely perceived as “bad mothers”, due to a criticism of “unhealthy” and “bad influences”, compared to their non-smoking counterparts [27]. This may result in false responses. However, in our study, it is unlikely that the respondents provided any false responses, since all participants volunteered to take part in this field study. The local doctors lived in the same village for a long time, and were very familiar with the participants. They were trained and were responsible for both collecting information from interviews, as well as collecting urine samples in the first, second, and third trimesters. Moreover, pregnant women in this study were found to have a higher risk of ETS exposure based on UC verification, especially in rural areas where tobacco is produced, when compared with other findings [13,28]. UC verification objectively shows the rate of ETS exposure for pregnant non-smokers. In China, a ban on smoking in any indoor public facility was instigated in 2011, and recently, a promotion of smoke-free homes began. Nevertheless, most smoke-free families only reside in urban areas or cities. It was difficult to carry out a smoking ban in homes or promote smoke-free families in rural areas, due to traditional norms, such as respecting elders and the male-centric nature of the home, where smoking is regarded as normal behavior which is unlikely to be forbidden, even if pregnant women and children are present. Therefore, health practitioners can mitigate the negative effects of prenatal ETS exposure by educating pregnant women about secondhand and thirdhand tobacco smoke when exposure to ETS is detected. Our findings confirmed those of a prior study which concluded that the number of smokers in the household was correlated with higher discrepancy rates between SR and UC verification [25]. As the number of smokers in the home increases from one to two or three smokers, the level of the discrepancy increases sharply. Thus, we could postulate that pregnant women tend to consciously or subconsciously ignore the negative effects of passive smoking, and accept ETS from smokers when there are more smokers in their families, such as husbands or grandfathers. The number of smokers in a family may result in a significant underestimation of SR passive smoking among pregnant women.

The annual household income was also found to have a statistically significant association with the discrepancy between SR and UC-verified rates of ETS exposure throughout pregnancy. The likelihood of a discrepancy between SR and UC-verified ETS exposure was found to increase as annual household income increased from CNY 15,000 to CNY 50,000, particularly when the household income increased beyond CNY 30,000. However, the odds of inconsistency decreased as the household income increased beyond CNY 50,000, indicating a nonlinear relationship (inverted V-shaped) between annual household income and discrepancy. Furthermore, our analysis showed that Township 1 was associated with a discrepancy between SR and UC-verified rates following the second pregnancy. Pregnant women from Township 1, an industrial township, demonstrated lower rates of both SR and UC-verified ETS exposure than Township 2, which was a tobacco-producing region. In addition, a gravidity of three and above was only associated with discrepancy in the last trimester. It could be said that the experience of pregnant non-smokers who are multigravida tend to not increase their awareness of passive smoking before delivery.

Some issues should be considered in this study. Non-respondents included those who had not answered specific questions in the questionnaire (item non-respondents) and those who did not provide three urine samples throughout their pregnancies, whereby participants missed the opportunity to collect urine when the surveys were conducted at the three time points during the pregnancy (sample non-respondents). In our study, the non-response rate for annual household income was considered low and acceptable, since it was quite low (5.2%), and the sample of non-respondents was excluded prior to our analysis. Moreover, participants in this study may be limited to the nature of the non-smoking pregnant women. In addition, our study potentially had a selection bias, as participants have relatively low household incomes and education in China. Finally, UC levels can be elevated with active smoking, nicotine replacement therapy, and electronic-cigarette use [29]. Though the participants in the study were non-smokers, their UC levels could be influenced by the number of cigarettes they were exposed to inside and outside the home, as well as to the indoor environment of rural homes, and the season of flue-cured tobacco owing to China’s main tobacco-producing region. In the future, a combinational analysis including the cut-off value of UC detection and the number of cigarettes can help evaluate the discrepancy.

To the extent of our knowledge, this is a novel study that obtained measures at three time points throughout a pregnancy from a prospective prenatal cohort to assess the discrepancy between SR passive-smoking rates and UC-verified passive-smoking rates, and to identify predictors of the discrepancy for each trimester of pregnancy. Also, we established that the number of smokers at home during the entire pregnancy, and gravidity (in the last trimester) may increase the risk of ETS exposure for rural pregnant women with a low socio-economic status.

## 5. Conclusions

In conclusion, the rate of ETS exposure reported by rural women with a low socio-economic status is underreported, and the UC-verified rate of ETS is higher during pregnancy. These subjective false replies impede the accurate assessment of ETS exposure among non-smoking pregnant women. Thus, we suggest that researchers should focus on the underlying and amplified discrepancy between SR and UC-verified ETS exposure when confronting more smokers in a family. The number of smokers in a family should be one of the important confounders in a questionnaire for pregnant women who are exposed to ETS. Qualitative research should be conducted among pregnant women to further understand factors influencing response rates. Furthermore, a higher number of pregnancies may increase the risk of pregnant women being passive smokers. The findings suggest that it may be useful for researchers to consider how different measures of passive smoking may influence their results. Biochemical validation is warranted throughout pregnancy to encourage smoking cessation and smoking bans in homes, and to encourage the development of community-based smoke-free programs in order to inform pregnant women and their communities of the importance of not smoking in the home and other indoor places, particularly when pregnant women are present.

## Figures and Tables

**Table 1:** Demographic characteristics for the study sample of rural pregnant women (*N* = 420).

Variable	Township 1 (*n* = 181)		Township 2 (*n* = 239)		Total	
	*n*	%	*n*	%	*n*	%
Age						
15–19 years	27	14.92	27	11.30	54	12.86
20–25 years	93	51.38	118	49.37	211	50.24
26–30 years	37	20.44	60	25.10	97	23.10
31–35 years	18	9.94	24	10.04	42	10.00
36 years and above	6	3.31	10	4.18	16	3.81
Ethnic groups						
Han	152	83.98	204	85.36	356	84.76
Non-Han	29	16.04	35	14.64	64	15.24
Occupation						
Farmer	119	65.75	217	90.79	336	80.0
Non-farmer	62	34.25	22	9.21	84	20.0
Education level						
6 years and below	73	40.33	68	28.45	141	33.57
7–9 years	83	45.86	136	56.90	219	52.14
10 years and above	25	13.81	35	14.64	60	14.29
Husband’s ethnicity						
Han	157	86.74	212	88.70	369	87.86
Non-Han	24	13.26	27	11.30	51	12.14
Husband’s occupation						
Farmer	81	45.25	209	87.45	290	69.38
Non-farmer	98	54.75	30	12.55	128	30.62
Husband’s education level						
6 years and below	54	30.0	49	20.50	103	24.58
7–9 years	90	50.0	163	68.22	253	60.38
10 years and above	36	20.0	27	11.30	63	15.04
Household income (past year)						
15,000 CNY and below	53	29.28	69	28.87	122	29.05
15,001–30,000 CNY	93	51.38	51	21.34	144	34.29
30,001–50,000 CNY	12	6.63	31	12.97	43	10.24
50,001 CNY and above	6	3.31	83	34.73	89	21.19
No response	17	9.39	5	2.09	22	5.24

**Table 2:** Differences in study variables between self-reported (SR) and urinary cotinine (UC)-verified ETS exposure (in percentages).

Variable	First Trimester			Second Trimester			Third Trimester		
	SR (%)	Cot. (%)	*p*-Value ^a^	SR (%)	Cot. (%)	*p*-Value	SR (%)	Cot. (%)	*p*-Value
Place									
Township 1	65.19	96.13	<0.001	72.93	98.34	<0.001	65.75	97.79	<0.001
Township 2	85.77	98.33		91.21	99.58		91.63	99.16	
Age									
15–19 years	79.63	100.0	0.148	83.33	100.0	0.935	74.07	94.44	0.373
20–25 years	72.04	97.16		82.94	99.05		80.57	99.53	
26–30 years	81.44	95.88		84.54	98.97		80.41	97.94	
31–35 years	80.95	97.62		80.95	97.62		83.33	100.00	
36 years and above	93.75	100.00		87.50	100.00		93.75	100.00	
Ethnic groups									
Han	77.53	97.19	0.962	83.99	99.16	0.711	79.78	98.60	0.479
Non-Han	73.44	98.44		79.69	98.44		84.38	98.44	
Occupation									
Farmer	78.87	97.02	0.103	83.63	99.11	0.605	82.14	98.51	0.251
Non-farmer	69.05	98.81		82.14	98.81		73.81	98.81	
Education level									
6 years and below	78.01	97.16	0.208	82.98	98.58	0.018	80.85	99.29	0.079
7–9 years	74.43	96.80		79.91	99.54		77.63	98.17	
10 years and above	83.33	100.0		96.67	98.33		90.00	98.33	
Husband’s occupation									
Farmer	80.34	97.93	0.002	85.86	99.31	0.012	83.79	98.97	0.011
Non-farmer	68.75	96.09		77.34	98.44		72.66	97.66	
Husband’s education level									
6 years and below	76.70	95.15	0.728	82.52	97.09	0.906	79.61	99.03	0.518
7–9 years	77.47	97.63		83.40	99.60		79.84	98.02	
10 years and above	74.60	100.00		84.13	100.00		84.13	100.00	
Household income (last year)									
15,000 CNY and below	74.59	95.90	<0.001	86.89	100.00	<0.001	81.15	98.36	<0.001
15,001–30,000 CNY	68.75	98.61		75.00	98.61		72.92	97.92	
30,001–50,000 CNY	72.09	95.35		72.09	97.67		76.74	97.67	
50,001 CNY and above	95.51	98.88		95.51	100.00		95.51	100.00	
No response	77.27	95.45		90.91	95.45		72.73	100.00	
Women’s awareness of passive smoking									
Yes	75.28	97.19	0.366	83.71	99.16	0.622	75.84	98.31	0.027
No	77.64	97.89		82.70	98.88		83.54	98.73	
Number of pregnancies									
1	79.69	99.22	0.101	85.94	100.00	0.080	82.03	99.22	0.051
2	77.60	97.27		83.61	98.91		83.06	97.81	
3 and above	70.93	95.35		76.74	97.67		70.93	98.84	
Number of smokers in the family									
0	93.55	97.58	<0.001	96.32	98.53	<0.001	89.12	97.96	<0.001
1	75.65	96.37		80.00	98.97		80.66	98.34	
2–3	59.22	99.03		70.79	100.00		66.30	100.00	
Total ^b^	76.90	97.38		83.33	99.05		80.48	98.57	

SR, self-reported; Cot., urinary cotinine; ^a^ based on the chi-squared test; ^b^ based on the chi-squared test for three trimesters, Pearson χ^2^ = 8.3954, *p* = 0.015.

**Table 3:** Factors associated with the discrepancy between SR and UC-verified ETS exposure among rural pregnant women.

Variable	First Trimester		Second Trimester		Later Pregnancy	
	Crude OR (95% CIs)	Adjusted OR ^a^ (95% CIs)	Crude OR (95% CIs)	Adjusted OR (95% CIs)	Crude OR (95% CIs)	Adjusted OR (95% CIs)
Place						
Township 2	1.00	1.00	1.00	1.00	1.00	1.00
Township 1	1.49 (0.75–2.96)	1.64 (0.81–3.34)	3.25 (1.41–7.50) ^b^	3.72 (1.56–8.88) ^b^	3.53 (1.68–7.42) ^b^	3.51 (1.65–7.47) ^b^
Husband’s occupation						
Farmer	1.00	1.00	1.00	1.00	1.00	1.00
Non-farmer	1.71 (0.92–3.19)	1.58 (0.83–3.00)	1.20 (0.61–2.37)	1.15 (0.57–2.33)	1.04 (0.54–1.99)	1.09 (0.57–2.11)
Household income last year						
15,000 CNY and below	1.00	1.00	1.00	1.00	1.00	1.00
15,001–30,000 CNY	1.14 (0.60–2.17)	1.20 (0.63–2.31)	2.33 (1.09–4.98) ^c^	2.40 (1.11–5.21) ^c^	1.63 (0.83–3.21)	1.56 (0.79–3.09)
30,001–50,000 CNY	1.12 (0.46–2.73)	1.25 (0.50–3.09)	4.95 (1.76–13.92) ^b^	5.09 (1.77–14.68) ^b^	2.13 (0.82–5.51)	2.07 (0.79–5.42)
50,001 CNY and above	0.11 (0.04–0.34) ^b^	0.11 (0.04–0.36) ^b^	0.47 (0.13–1.77)	0.41 (0.11–1.59)	0.27 (0.08–0.94) ^c^	0.28 (0.81–0.98) ^c^
Number of pregnancies						
1	1.00	1.00	1.00	1.00	1.00	1.00
2	1.22 (0.63–2.37)	1.42 (0.70–2.87)	1.09 (0.52–2.28)	1.07 (0.48–2.35)	1.15 (0.57–2.29)	1.37 (0.65–2.88)
3 and above	1.96 (0.89–4.33)	2.32 (0.93–5.79)	2.36 (1.02–5.46)	1.96 (0.73–5.27)	2.11 (0.95–4.68)	2.72 (1.08–6.81) ^c^
Number of smokers in the family						
0	1.00	1.00	1.00	1.00	1.00	1.00
1	7.73 (2.88–20.74) ^b^	7.14 (2.64–19.31) ^b^	5.29 (2.04–13.69) ^b^	5.01 (1.94–12.96) ^b^	1.65 (0.82–3.33)	1.56 (0.76–3.17)
2–3	25.71 (8.94–73.94) ^b^	24.22 (8.35–70.26) ^b^	11.78 (4.22–32.86) ^b^	12.49 (4.45–35.05) ^b^	5.96 (2.75–12.91) ^b^	6.10 (2.78–13.39) ^b^

^a^ OR, odds ratio; CIs, confidence intervals; estimated by logistic regression and adjusted for the pregnant women’s age and awareness of passive smoking. ^b^ *p* < 0.01; ^c^ *p* < 0.05.
